# Drivers of transformation of the maize sector in Nigeria

**DOI:** 10.1016/j.gfs.2023.100713

**Published:** 2023-09

**Authors:** Tesfamicheal Wossen, Abebe Menkir, Arega Alene, Tahirou Abdoulaye, Sam Ajala, Baffour Badu-Apraku, Melaku Gedil, Wendie Mengesha, Silvestro Meseka

**Affiliations:** a International Institute of Tropical Agriculture, Nairobi, Kenya; b International Institute of Tropical Agriculture, Ibadan, Nigeria; c International Institute of Tropical Agriculture, Bamako, Mali

**Keywords:** Certified seed, Food security, Improved seed, Maize, Nigeria

## Abstract

Maize is widely used for food, animal feed, and industrial raw material in Nigeria. This paper documents the important changes that characterize Nigeria's maize production and area expansion along with contributing factors that have transformed maize from a backyard food crop to a dominant food security and commercial crop. Using both secondary and primary data on maize production and varietal adoption over the last six decades, we found that Nigeria now produces ten times more maize than it did in 1960 and four times more maize than it did in 2005. Our findings further suggested that government policies and institutional arrangements that promoted access to and use of modern inputs and increased demand of maize grain for food, feed, and other industrial uses have played major roles in transforming maize from a backyard crop to a dominant staple and commercial crop in Nigeria. Considering the impeding climate change threats to food security in Nigeria, policy interventions should be tailored towards further scaling-up of stress resilient and climate-smart maize varieties to improve the productivity, income, and resilience of smallholder farmers. This requires strong support not only to get recently released superior improved varieties into the hands of smallholder farmers but also to accelerate varietal turnover.

## Highlights

• Maize is widely used for food, animal feed, and industrial raw material in Nigeria.
• Nigeria’s maize production growth is driven by both area expansion and adoption of yield enhancing modern inputs.
• Rapid growth in maize grain demand for processed food, feed, and other industrial uses is an important driver of maize production growth in Nigeria.
• Scaling stress resilient improved maize varieties is key to improve smallholders’ food security and climate resilience.

## 1 Introduction

Maize is a crop of notable interest for food security in many parts of sub-Saharan Africa (SSA) (van Ittersum et al., 2016; Fisher et al., 2015; Foltz et al., 2012). As in other countries of SSA, maize is an important crop in Nigeria, where it is largely cultivated by smallholder farmers over 6.5 million hectares of land across diverse agroecological zones of the country (Onumah et al., 2021; pwc, 2021; FAOSTAT, 2022). It is by far the largest cereal crop in terms of area and production volume and is the most consumed staple food in Nigeria (Onumah et al., 2021). In Nigeria, maize is widely used for human consumption, in animal feed, pharmaceutical industries, food manufacturers, breweries, flour mills and other industries. Nearly 80 percent of the maize grain is used for human consumption and animal feed with the remaining 20 percent utilized for industrial processing of diverse products (Onumah et al., 2021). With a per capita consumption of about 35 kg per person per year, maize accounts for an estimated 10% of the daily calorie intakes in the country*.* The crop is also an important source of cash income for farmers and contributes significantly to agro-industrial development particularly in the livestock feed industry (Alene et al., 2009; Wossen et al., 2017b).

Following its arrival in West Africa in the 15th century (Miracle, 1965), maize became an invaluable crop fitting into the existing diverse farming systems because of its broad adaptation to varying growing conditions, ease of processing and resistance to pre-harvest bird damage that plagued sorghum, millet, and rice (McCann, 2005; Fakorede et al., 2022). As a quick maturing crop, it has become a critical source of food for rural families when food reserves are depleted before the root crops, sorghum, millet, and other native crops are harvested. Until the 1970s, maize was mainly grown in the humid forest agro-ecology as a source of fresh maize for boiling and roasting, and grain for processing and conversion into diverse local food products (Fajemisin, 2014). It is now cultivated even in the drier areas which are traditional niches for sorghum and millet due to the development and commercialization of drought tolerant extra-early and early maturing maize varieties that provide food when grain reserves are depleted after the long dry period because of their faster maturity cycle and better responsiveness to fertilizer compared to sorghum and millet (Badu-Apraku et al., 2015; Fajemisin, 2014).

Until the mid to late 1970s, maize production in the Nigerian savanna was limited to homesteads. However, as demand for maize grain for food, animal feed, and other industrial products increased, significant expansion in maize production occurred in the savannas where it became a prominent commercial crop competing with other indigenous cereals (Smith et al., 1994). The spread of maize to this region was spearheaded by its high yield potential due to adequate rainfall, long growing period, dry weather at harvesting time and low disease and insect pressure (Kassam et al., 1975) coupled with the availability of high yielding and adapted maize varieties and supportive government programs that promoted increased local production (Fajemisin, 2014). These conditions led to the phenomenal increase in maize production and established the Nigerian savannas as the major supplier of maize for food and other uses. The introduction of high yielding and stress resilient maize varieties combined with many years of investment by the Federal Government of Nigeria, continuous involvement of a multitude of research, development, financial and other institutions, and seed and input suppliers as well as supportive government policies catalyzed this impressive progress in maize production (Fajemisin, 2014; Fakorede et al., 2022). Maize has thus achieved a prominent status in Nigeria's food system and established itself as a crop well integrated into the national economy and local diets notwithstanding the presence of enormous diversity in test, traditions, aesthetic choices, and agro-climatic zones. With maize production exceeding 12 million MTs in recent years, Nigeria currently ranks second in Africa and, as a result, has emerged as one of the leading maize producers in the world (Onumah et al., 2021).

While the current yields in Nigeria of around 2 tons per hectare are much lower than what can be attainable with the available improved production technologies and suitable production environments, the rate of production and area expansion has been remarkable over the last 60 years. What are the key contributing factors to the significant expansion of maize production in Nigeria? How much is due to expanded use of inputs such as improved seed and fertilizers versus area expansion? In an attempt to address these questions, this paper analyzes and synthesizes available evidence on maize production, productivity and varietal adoption over the last six decades. More specifically, using both secondary and primary data sources, including nationally representative household surveys, this paper provides descriptive evidence on the drivers of maize sector transformation including (i) commercialization of maize to meet increased demand for food, feed and industrial use, (ii) accelerated release of stress resilient, productive and adapted maize varieties coupled with increased availability of certified seeds to farmers, (iii) the establishment and changing landscape of the private sector, and (iv) supportive government policies that stimulated accelerated production of maize in the country by facilitating farmers’ access to and use of productivity-enhancing inputs such as improved seed and fertilizer. Finally, using DNA-Fingerprinting based varietal identification, the paper provides descriptive evidence on varietal traits associated with greater adoption in Nigeria.

## 2 Materials and methods

We used different datasets to assess trends in maize area, production, yield, adoption of productivity-enhancing inputs such as chemical fertilizer and improved seed in Nigeria. In particular, we relied on data from two sources: (1) secondary data sources including past studies, public policy documents, government statistics, and project documents; and (2) primary data from nationally representative farm and household level variety monitoring and adoption surveys. Although several national and state level agencies provide agricultural production related statistics for Nigeria, these data are often not comprehensive and nationally representative. Hence, for our main analysis, we compiled comprehensive long term national level production, area and yield estimates from the Wet Season Agricultural Performance Survey Reports of the National Agricultural Extension and Research Liaison Services (NAERLS) (www.naerls.gov.ng) and obtained additional production statistics from the Nigerian National Bureau of Statistics (NBS) (NBS, 2007; NBS, 2011). These sources of data are related to smallholder farmers’ production during the major wet growing seasons in Nigeria. We also compiled long term national level production, area and yield estimates from the Food and Agriculture Organization of the United Nations (FAOSTAT, 2022) and the USDA Production, Supply, and Distribution (USDA-PSD) online database for comparative assessment. Moreover, we reviewed the government of Nigeria economic and agricultural statistics, project descriptions from development partners, international development organizations, and non-governmental organizations to provide additional insights into the transformation of the maize sector in Nigeria.

To complement the NAERLS, NBS and FAO data, we compiled additional micro-level data from large-scale and nationally representative farm and household surveys. These included: (a) the Nigeria Country Plan Baseline and Varietal Monitoring survey (NIBAS, 2017), (b) the project survey for the Drought Tolerant Maize for Africa (DTMA, 2014) and (c) the Nigerian Living Standards Measurement Study-Integrated Surveys on Agriculture (LSMS-ISA, 2018). These data are nationally representative and provide comparable statistics across space and time. The NIBAS survey was conducted across six states in Nigeria, including Benue, Kaduna, Kano, Katsina, Nasarawa, and Niger that are representative of the maize belt of the country. Similarly, the DTMA survey was conducted across 18 major maize producing states in the country, and thus represented the maize farming households in Nigeria. Both the NIBAS and DTMA surveys provided data on varietal choice, source of improved seed, seed purchasing history, trait preferences, yield and other key socio-economic characteristics from a nationally representative sample of approximately 3000 maize growers in Nigeria. To circumvent traditional survey-based measurement errors in estimating varietal adoption, NIBAS used a novel DNA-fingerprinting based varietal identification to accurately identify maize varieties grown by farmers in Nigeria. We used the DNA-fingerprinting based varietal tracking information to document the extent of maize area planted with improved maize varieties and the area-weighted average age of the maize varieties grown in the country.

## 3 Results

### 3.1 Trends in maize area, yield, and production in Nigeria

**Fig. 1:**
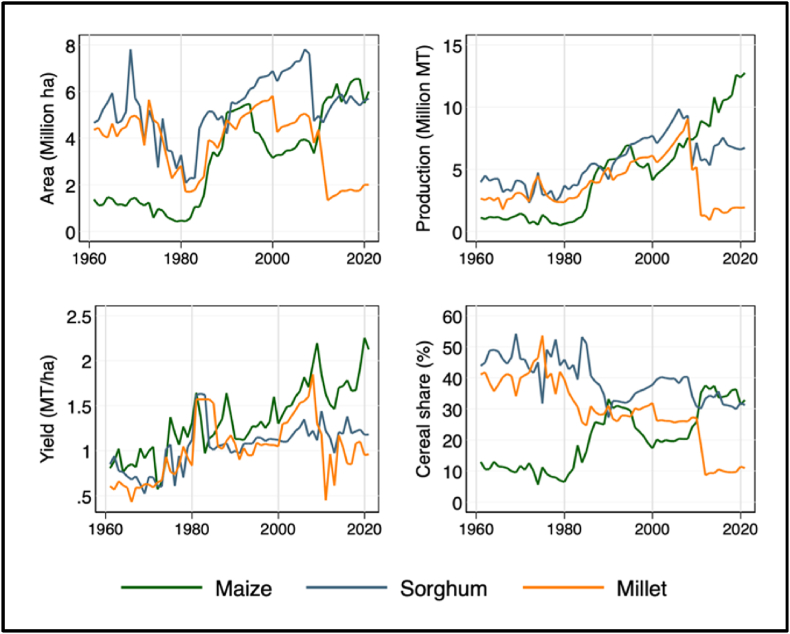
Production, area and yield trends of maize, millet, and sorghum.

Source: Authors calculation based on FAOSTAT

As shown in Fig. 1, Nigeria recorded impressive growth in maize production over the last 60 years (FAOSTAT, 2022). The total maize production consistently exceeded 10 million MTs in the past few years, making Nigeria one of the leading maize producers in Africa. With maize production exceeding 12 million MTs in recent years, Nigeria has become the second largest maize producing country in Africa. The country is also the leading producer of maize in West Africa, accounting for more than 48% of the total maize output in the region between 2015 and 2020.

From 1961 to 2020, maize production and harvested area increased by 984% and 450%, respectively. Holding input and land use constant, Nigeria now produces ten times more maize per year than it did in 1960. As shown in Fig. 1, significantly smaller land area was cultivated to maize relative to both sorghum and millet in the 1960s and 1970s, but the area under maize cultivation increased substantially in the 1980s. The increased availability of disease and pest resistant improved maize varieties along with fertilizer subsidies played a key role in the significant expansion of maize cultivation into the savannas of Nigeria (Smith et al., 1994; Alene et al., 2009; Byerlee and Jewell, 1997; Byerlee, 2020). During this period, growth in maize production was mainly driven by extensification in the land-abundant savannas that were characterized by extensive fallow periods (Byerlee and Jewell, 1997; Byerlee, 2020).

**Fig. 2:**
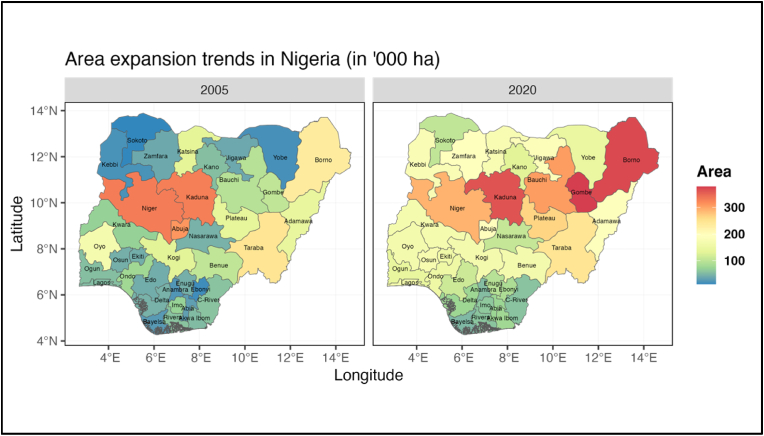
Spatial and temporal dimension of maize production expansion in Nigeria.

Source: Author's calculation based on NBS and NAERLS data

**Fig. 3:**
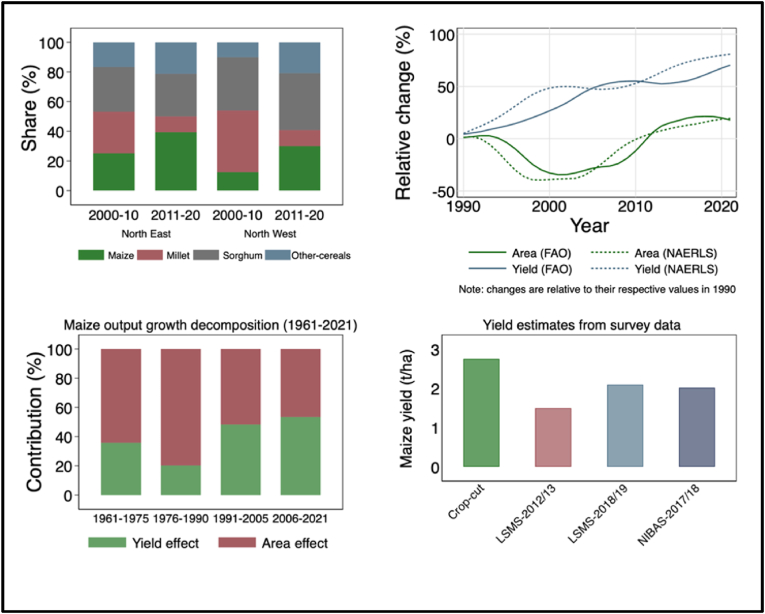
Maize production, area and yield growth in Nigeria.

Source: Author's calculation based on FAOSTAT, NBS and NAERLS data

Since the 1980s, the steady growth of maize production has coincided with several maize research initiatives in the country that have led to the development and release of suitable maize varieties by the national partners in collaboration with IITA (Kassam et al., 1975). What is even more impressive is the increase in maize area relative to other major cereals in the country. Maize occupied roughly 16% of the total cereal area in 2000 and its share increased to about 33% in 2020, overtaking sorghum as the most widely grown cereal crop since 2011. Fig. 3 (top left graph) shows that while the area under cereal cultivation has remained the same, the area under maize cultivation increased significantly at the expense of other cereals especially millet. Therefore, maize area expansion has been mostly driven by the replacement of millet and sorghum and not through expansion into forest and grasslands. The maize area expansion in Nigeria can thus be attributed to both expansion into new areas in the north-western and north-eastern regions of the country where the short rainy season hitherto had precluded maize cultivation and the displacement of millet and sorghum with maize having higher yield and market potential (see Fig. 2).^1^

The relative changes in maize area expansion and yield improvements over the period from 1990 to 2020 (top right graph of Fig. 3) shows that maize yields have increased at a higher or similar rate relative to cultivated area, suggesting that maize production growth was driven by both yield improvements and expansion of area under cultivation. Between 1990 and 2020, yield and area under maize cultivation increased by 34 and 42%, respectively. These growth rates in maize production using FAO data are largely consistent with the growth rates found using alternative international and national level production statistics from USDA-PSD and NAERLS that show an increase in maize production from about 4 million MT in 1990 to 12 million MT in 2020. Between 2010 and 2020 alone, maize production and its cultivated area increased by about 4.2 and 0.43% per year, respectively. During the same period, maize yields grew by an average of 3.9% per year which was slightly higher than the growth rate computed from the FAO data. Based on the USDA-PSD data, maize yields, and area growth rates between 2015 and 2020 were 9.6% and 3.6%, respectively.

To provide a broader perspective, we decompose the source of maize production growth into effects attributable to yield and area using the Shapley decomposition of the total explained variation in maize production growth (bottom left graph in Fig. 3). We find that between 1961 and 1990, maize output growth was driven largely by area expansion and much less by productivity growth: the shapely decomposition results suggest that area expansion explained close to 70% of the total variance in maize output growth between 1961 and 1990. Since 1991, maize output growth has been driven by both area expansion and productivity growth, which is reflected in the respective 52 and 48 percent contribution of yield and area effects. The growing contribution of yield to output growth may reflect greater access of farmers to modern inputs such as fertilizers and improved seeds. For instance, before improved maize varieties were available in the 1990's, the average maize yield in Nigeria was only half of the estimated maize yield in 2020.

One concern that arises with the use of aggregate national level statistics is that it may be contaminated by measurement error and hence data may simply reflect inaccurate reporting. To collaborate the above national level productivity change trends in maize, Fig. 3 (bottom right graph) reports maize yields using nationally representative crop-cut data collected from more than 3000 plots in Nigeria and farmer-recall based production data from the Nigerian Living Standards Measurement Study-Integrated Surveys on Agriculture (LSMS-ISA, 2018/19). Two results are worth highlighting: First, all the micro-level yield estimates are largely consistent with the FAO estimates. Second, the mean yields of maize are considerably higher when measured with crop cuts, as compared with both farmer-recall based yield measures from micro-level data and the aggregate yield measures from FAO and NAERLS. The difference in the two conditional means is statistically significant at any reasonable significance level.

### 3.2 Drivers of maize production growth in Nigeria

The evidence presented in the previous section illustrates the important changes that characterize Nigeria's maize production and area expansion in the last decades. The major potential drivers underlying maize production and area expansion in the country are discussed below.^2^ The first set of drivers linked production growth with adoption of yield enhancing inputs, with a focus on improved seed and chemical fertilizers. The second set of drivers linked production growth with a shift in maize grain demand for processed food, feed, and other industrial uses.

#### 3.2.1 Favorable government policies and institutional arrangements

**Fig. 4:**
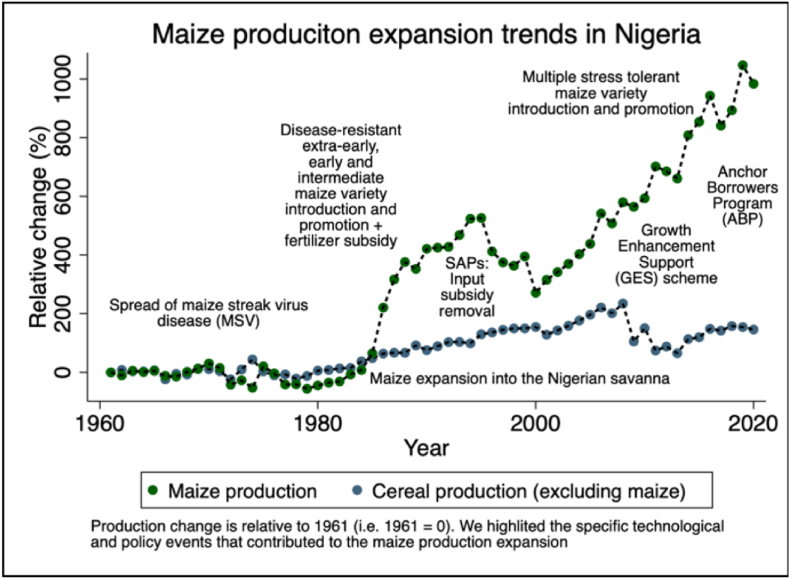
Selected drivers of maize production growth and area expansion in Nigeria.

Source: Author's calculation based on various data sources.

Government policy plays an important role in fostering agricultural productivity growth and rural transformation (Foltz et al., 2012). The Federal government of Nigeria has made several important policy changes and introduced support programs that promoted the use of improved seeds, fertilizer, and other complementary farm inputs, including many supply side value chain development interventions that played prominent roles in transforming the maize sector. A summary of the major events that significantly shaped the evolution of Nigeria's maize sector are presented in Fig. 4. Accelerated growth started in the mid-1980s following the introduction of maize varieties resistant to the maize streak virus, downy mildew, southern corn leaf rust, and southern corn leaf blight as well as access to subsidized fertilizer, improved extension services and infrastructure (Smith et al., 1997). The promotion of particularly early and extra-early maturing maize varieties that mature in 80–95 days enabled the expansion of maize into the dry savannas of the country with short growing periods leading to the rapid replacement of millet and sorghum (Badu-Apraku et al., 2015). During this period, the government launched an Operation Feed the Nation (OFN) program that promoted seed and fertilizer use through subsidies. This was followed by the National Accelerated Food Production Program (NAFPP), the National Agricultural Extension and Research Liaison Services (NAERLS) and the Agricultural Development Projects (ADPs) all playing key roles in the expansion of maize production into the savannas. As part of these programs, more than 3000 maize varietal mini-kit trials were conducted all over the country to promote the use of improved maize varieties, fertilizer, herbicides, and other appropriate crop management practices (Fajemisin, 2014). However, maize production stagnated in the 1990s primarily due to the removal of fertilizer subsidies as part of the structural adjustment program (SAP).

In 2006 the Presidential Initiative on Doubling Maize Production (PIDOM) was launched due to the staggering demand of maize grain for human consumption, poultry feed and industrial use (Fajemisin, 2014; Olomola, 2015). The focus of the PIDOM was a private sector driven approach with substantial support from the government to research, input subsidy, providing guaranteed minimum grain price to farmers and a grain buy-back policy to encourage production. With the support of the PIDOM and the Drought Tolerant Maize for Africa (DTMA) project, more than 25 new stress tolerant and high yielding maize hybrids and open-pollinated varieties were released and promoted to farmers across all the major maize producing states of the country. These varieties were then widely promoted to mitigate the impact of combined multiple stresses affecting maize farming, including, *Striga*, drought, and poor soil fertility contributing to the phenomenal increase in maize production in recent years.

In 2011, the 10.13039/100015539Federal Government of Nigeria launched the Agricultural Transformation Agenda (ATA) and introduced a Growth Enhancement Support (GES) scheme with the aim of increasing the productivity of smallholders through a mobile phone based targeted subsidy (Olomola, 2015; Wossen et al., 2017a; Fakorede et al., 2022). The GES provided a 50% subsidy on two bags of fertilizer and up to 90% subsidy on a bag of improved maize and rice seeds. In doing so, the GES contributed immensely to the promotion and commercialization of multiple stress tolerant maize varieties developed with the support of the DTMA and the Stress Tolerant Maize for Africa (STMA) projects. For instance, the GES facilitated the distribution of fertilizer and improved seeds to more than 4.1 million farmers in 2013 and 7.2 million farmers in 2014 (Fajemisin, 2014; Olomola, 2015).^3^ The commercialization of these varieties accelerated the expansion of maize into new areas where the occurrence of stresses hitherto had precluded maize cultivation in the past.

#### 3.2.2 Accelerated development and release of stress resilient and productive varieties

The development and deployment of improved crop varieties is crucial to realize productivity gains from genetic improvements. In this regard, the number of varieties released by the national breeding programs can be indicative of the performance of the maize sector through enhanced farmers’ access to improved seed. The national agricultural research systems (NARS) in Nigeria started breeding maize in the 1950s and released 16 maize varieties between 1950 and 1975 (NACGRAB, 2014). With the establishment of the International Institute of Tropical Agriculture (IITA) in 1967, a more rigorous approach was pursued to improve maize in partnerships with the NARS in Nigeria (Fakorede et al., 2022). Driven by the national demand for hybrid maize seed, IITA and its national partners embarked on hybrid development in 1979 with the financial support from the Federal Government of Nigeria that led to the release of the first high yielding and adapted hybrids in 1983. With consistent financial support from the international donor agencies and the Federal Government of Nigeria, the strong partnership between the NARS and IITA established over the years spearheaded the efforts made to develop and deploy new maize hybrids and open-pollinated varieties that are more productive, tolerant to drought, and resistant to diseases and *Striga* (Fajemisin, 2014). These investments led to the development and release of 124 open-pollinated maize varieties and hybrids from 1981 to 2020. The national agricultural research institutes in close collaboration with IITA and private seed companies were responsible for the registration and release of these varieties in Nigeria.

**Fig. 5:**
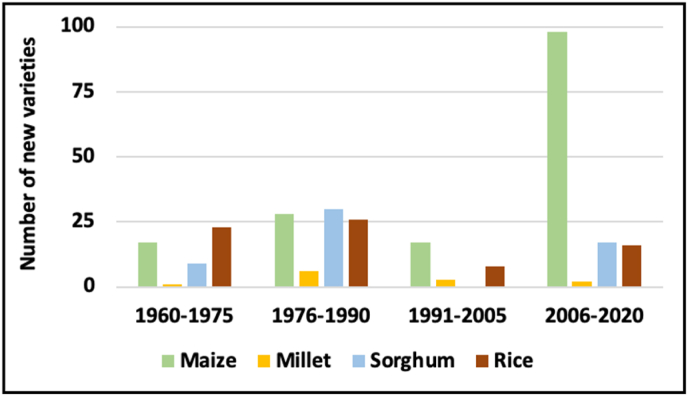
Trends in new variety releases (1960–2020).

Source: Author's calculation based on various data sources.

Prior to 2007, 37 open pollinated maize verities (OPVs) and 25 hybrids were released in Nigeria, representing 44% of the total number of released varieties. As shown in Fig. 5, Nigeria witnessed accelerated variety releases between 2006 and 2020, which coincided with the periods of implementations of the DTMA and STMA projects, and the launch of the PIDOM. Amongst the stress resilient and other maize varieties released during this period, 36 were hybrids and 42 were OPVs. Most of the OPVs and hybrids showed yield advantages of 20–30% over commercial hybrids or the current farmer preferred improved OPVs across stressful and favorable field conditions in Nigeria. Many of these maize OPVs and hybrids have been commercialized in Nigeria in close collaboration with the NARS and private seed companies. Indeed, the accelerated development and release of stress resilient and productive maize varieties has contributed to significant maize production growth achieved in Nigeria and will remain the cornerstone for further transformational impact as many released varieties and complementary technological innovations are available and can be readily deployed.

#### 3.2.3 Emergence of private seed companies

A competitive seed sector is critical to ensuring the timely availability of high-quality seeds of improved varieties to smallholder farmers at affordable prices (Waithaka et al., 2019; Mabaya et al., 2021). The successful development and release of the first high yielding and adapted hybrids in 1983 triggered the creation of the first Nigerian seed company for marketing hybrid maize. Soon after this, the favorable policies of the Federal Government of Nigeria encouraged the involvement of many institutions, individuals, multi-national and regional seed companies to venture into hybrid maize production and marketing in the country (Fajemisin, 2014). This led to the production and sale of close to 1000 tons of hybrid maize seeds every year in 1986 and 1987. Nonetheless, following the removal of fertilizer subsidies, the demand for hybrid maize seed declined significantly starting from 1990, leading to the closure of many seed companies (Fakorede, 2022).

**Fig. 6:**
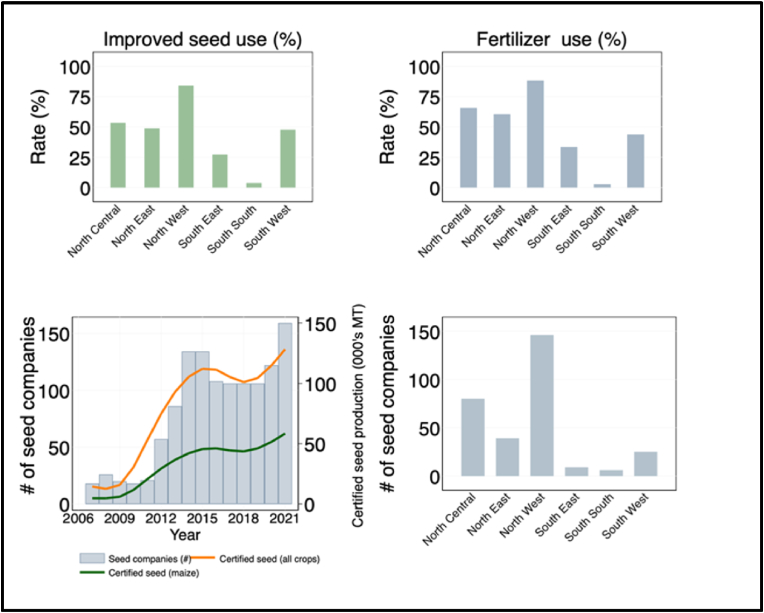
Role of seed companies and agro-dealers.

Source: Author's calculation based on various data sources.

As part of the Agricultural Transformation Agenda (ATA), the Federal Government of Nigeria introduced the GES scheme in 2011 to provide farmers with subsidized seeds of improved maize varieties and fertilizer (Fakorede et al., 2022; Wossen et al., 2017a). The ATA program focused on identification of a set of recommended improved maize varieties valued by farmers, production of adequate quantity of foundation seeds of these varieties by reliable agencies, and engagement of private seed companies to produce and deliver good quality certified seeds to smallholder farmers. A key feature underpinning the implementation of the GES was the liberalization of foundation and certified seed production by outsourcing the procurement and distribution of fertilizer and improved seeds to local private seed companies (Olomola, 2015; Wossen et al., 2017a). As a result of these, both the number of registered seed companies and the volume of certified seed production increased considerably (Fig. 6). The National Agricultural Seeds Council (NASC) reported that 21 seed companies that were actively involved in the production and marketing of maize seeds in 2011 plus additional 59 new seed companies were engaged in certified seed production in the first year of the GES program (Fajemisin, 2014).^4^ The number of seed companies participating in the production of certified seed under the GES scheme increased to 86 in 2013 and 134 in 2014 (NASC, 2013; NASC, 2014). As shown in Fig. 6, the availability of improved seed closely followed the establishment and engagement of these private seed companies. In Fig. 6 (upper panel), we also show how farmers’ access and use of improved seed and fertilizer is influenced by the spatial distribution and concentration of private seed companies and agro-dealers. As can be seen in Fig. 6, regions with greater adoption of improved seed and fertilizer also have greater concentration of input suppliers, consistent with the findings of Aggarwal et al. (2018) and Rutsaert et al. (2021) in Tanzania.

#### 3.2.4 Availability of certified seeds of broadly adapted improved varieties

It is widely recognized that the development, dissemination, and adoption of high yielding improved varieties is the primary pathway through which technological change in the agricultural sector can achieve productivity gains (Gollin et al., 2021; Wossen et al., 2019; Foltz et al., 2012). Evidence suggests that replacing low-quality hybrid maize seed with authentic, high-quality seed could provide productivity gains of over 80% (Bold et al., 2017). As farmers do not accurately report the identity of maize varieties they grow, we documented adoption rates of improved maize varieties using DNA-fingerprinting of samples collected from farmers' fields across six states in Nigeria (Wossen et al., 2019, 2022; Wineman et al., 2020). The results showed that at least 67% of the maize area in Nigeria is planted with improved maize varieties,^5^ whereas the traditional adoption survey based on farmers’ self-reports showed an adoption rate of 43%. The DNA-based study revealed that the most widely adopted improved maize varieties in Nigeria were the recently released stress tolerant OPVs such as SAMMAZ 15. These varieties are tolerant to multiple stresses which are essential for ensuring food security in rainfed production zones and semi-arid agro-ecologies. Wossen et al. (2017b) found that the cultivation of drought tolerant improved maize varieties reduced yield variability by 53% and the probability of food scarcity by 84%.

**Fig. 7:**
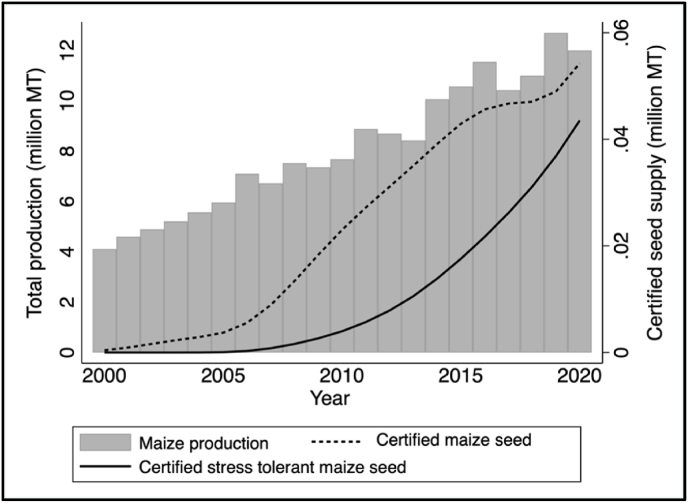
Production and marketing of certified maize seeds in Nigeria.

Source: Author's calculation based on various data sources.

Similarly, certified maize seed production in Nigeria increased from about 5314 MT in 2010/11 to over 60,000 MT in 2020/21 (Fig. 7). At the seeding rate of 25 kg/ha and assuming full utilization in the same production season, the estimated maize area covered with certified improved maize seed increased from about 0.22 million ha in 2010/11 to more than 2.4 million ha in 2020/21. The tenfold growth in certified seed production in the last 10 years resulted in an increase in cultivated maize area planted with certified seed from about 3% in 2010/11 to about 35% in 2020/21. Fig. 7 shows that the trends in maize production mirrors the trends in certified seed production, indicating that increased availability of improved seed is a major driver of maize production and productivity growth.^6^

**Fig. 8:**
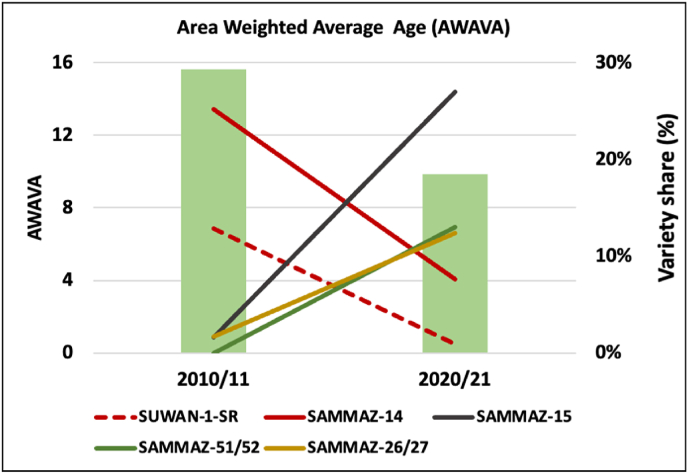
Maize varietal turn-over trends in Nigeria.

Source: Author's calculation based on various data sources.

Continual replacement of old varieties with new and superior varieties with high yield potential and tolerance to multiple stresses can maximize farmers' benefits and adaptation to changing climate (Chivasa et al., 2022). Such faster varietal turnover is expected to play an important role not only in realizing genetic gains in farmers’ fields but also in protecting against diseases and pests. The DNA-based assessment of the area-weighted average age of maize varieties (AWAVA) cultivated in the 2017/18 season in Nigeria was about 10 years, which is faster than the 2013/14 AWAVA estimate of 13 years reported by Abate et al. (2017). Similarly, the AWAVA values computed based on the total volume of certified maize seed production in Nigeria declined from 16 years in 2010/11 to 10 years in 2020/21. Such accelerated varietal turnover and large-scale adoption of improved varieties is a major driver of maize production and productivity growth (Evenson and Gollin, 2003; Alene et al., 2009). The trends in certified maize seed production and marketing in Nigeria suggest that the significant reduction in AWAVA has been largely driven by demand for stress tolerant varieties, including SAMMAZ 15, 51, 52. A key example of the AWAVA story in Nigeria is highlighted using two varieties, including SAMMAZ-15 released in 2008 and SUWAN-1-SR released in 1996 (Fig. 8). The share of SAMMAZ-15 from the total certified maize seed production in Nigeria increased from 1.6% in 2010/11 to about 30% in 2020/21, whereas the share for SUWAN-1-SR declined from about 13% in 2010/11 to less than 1% in 2020/21. Also, the share of SAMMAZ-14, which was released in 2005, declined from 25% in 2010/11 to about 7% in 2020/21, while the share of recently released varieties including SAMMAZ-51 and SAMMAZ-52 is increasing (Fig. 8).

#### 3.2.5 Promotion of complementary modern input use

**Fig. 9:**
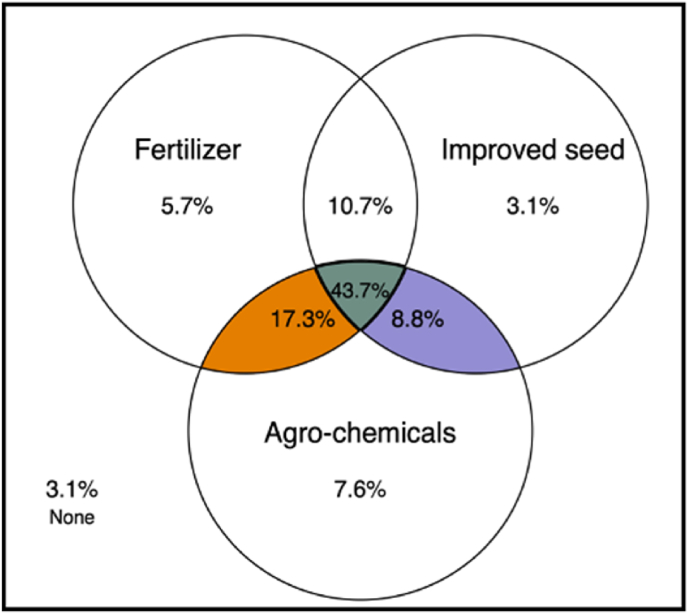
Adoption of complementary modern inputs among maize farmers.

Source: Author's calculation based on NIBAS (2017) and DTMA (2014).

It is commonly argued that modern farm-inputs are rarely adopted in isolation as adopting a basket of complementary inputs is advantageous for farmers (Sheahan and Barrett, 2014; Liverpool-Tasie et al., 2017; Byerlee et al., 2014). A combination of better varieties, crop management practices, and fertilizers is often considered important for realizing higher yield gains in farmers' fields. We thus examined maize farmers’ use of improved seed and inorganic fertilizer based on farm level data from the micro-datasets. As shown in Fig. 9, there is a significant overlap in the use of inorganic fertilizer, improved seed, and agro-chemicals among maize farmers in Nigeria. The overlapping area representing the use of at least two of the three inputs is considerably large. About 44% of the maize farmers use inorganic fertilizer, improved seed and agro-chemicals. At the same time, only about 12% of the farmers use improved seed without fertilizer, suggesting that there has been a spectacular increase in fertilizer use through adoption of yield-enhancing farm inputs as a bundle.

To quantify the contribution of complementary farm inputs to maize productivity in Nigeria, we examined the correlation between maize yields and use of improved seed and inorganic fertilizers based on the NIBAS and DTMA datasets. We use standard ordinary least squares (OLS) and instrumental variable (IV) regression approaches to estimate the association between adoption of improved maize varieties and maize yields. Estimates reported in Table 1 show a positive and statistically significant association between maize yields and the use of both improved seed and inorganic fertilizers. These results are consistent with the recent empirical evidence showing that improved seed confers a yield benefit (Abdoulaye et al., 2018; Wossen et al., 2017b, 2019). With about 927 kg/ha more maize grain obtained by cultivating improved varieties (based on the instrumental variable estimates reported in column 5), 1 kg of improved seed provides about 40 kg additional maize grain at the optimal seedling rate of 25 kg/ha that translate into an increase in maize grain production of 5 million MT per year. Although attributing productivity gains to adoption is difficult, both the crop-cut based national yield estimates reported in Fig. 3 and our exploratory correlation analysis in Table 1 suggests that the national maize production in Nigeria would have been lower by almost 50 percent without investments in improved variety development and deployment, input use, favorable policies and extension services.

**Table 1:** Correlation between adoption of modern inputs and maize yields.

	OLS			IV	
	1	2	3	5	6
Improved variety (yes=1)	746.4***	700.6***	514.72***	927.2***	0.533***
	(123.4)	(124)	(105.6)	(189.9)	(0.05)
Inorganic fertilizer (yes=1)		500.5***	282.2**		
		(108.9)	(123)		
Other controls	Yes	Yes	Yes	Yes	Yes
Number of observations	2817	2817	2817	2817	2817

*Notes*. The dependent variable in the first 5 columns is maize yield (kg/ha). In column 6, we use log-transformed maize yields as the dependent variable. Standard errors clustered at enumeration area level are given in parentheses. *p < 0.10, **p < 0.05, ***p < 0.01. Other controls include plot characteristics such as soil type, soil fertility, plot area, agro-chemicals (insecticide, pesticide and fungicide use at the plot level), mechanization, labor use, and location dummies are location dummies. In our instrumental variable (IV) regressions, we used self-reported improved variety indicator as an instrument for the DNA-based improved variety adoption indicator.

In Nigeria, adoption of improved maize varieties with varying maturity and drought tolerance could improve food security without significant productivity gains, particularly in marginal environments. This is possible particularly in the dry savannas of Nigeria where maize area expansion occurred through replacement of the less productive millet and sorghum varieties to improve overall food availability. Consistent with the findings of previous studies (Wossen et al., 2017b; Simtowe et al., 2019), estimates reported in Table 2 show that cultivation of multiple stress tolerant maize varieties significantly reduces food insecurity and the probability of experiencing seasonal food scarcity and hunger. Considering the impeding climate change threats to food security in Nigeria, policy interventions should thus be tailored to further scaling of seeds with multiple stress tolerance to build the resilience of smallholder farmers to climate shocks, increase maize productivity growth, and improve the welfare of farming households.

**Table 2:** Correlation between cultivation of multiple stress tolerant (ST) varieties and food security.

	HIFAS	Experience hunger	Eat inadequate quantity	Eat fewer meals
Cultivate ST varieties (yes)	−0.33**	−0.069***	−0.058***	−0.038*
	(0.147)	(0.026)	(0.018)	(0.022)
Other controls	Yes	Yes	Yes	Yes
N	2817	2817	2817	2817

*Note*: Standard errors in parentheses; ***P < 0.01, **P < 0.05, *P < 0.1. HIFAS denoted Household Food Insecurity Access Scale. ST varieties refers to growing stress tolerance improved maize varieties.

#### 3.2.6 Increased domestic demand for food, feed and industrial use of maize

In Nigeria, rapid growth in maize demand for food, feed and industrial uses has been an important driver of the dramatic growth in maize production in the last two decades (Dalberg, 2019). Driven by urbanization and income growth, a shift in demand for feed and processed food has provided growth stimulus for the maize sector in Nigeria. Industrially processed maize products for human consumption and flour millers, food manufacturers and breweries utilize up to 25% of the maize grain produced in the country (CONTEXT, 2015; Dalberg, 2019). Only about 11% of the maize grain in Nigeria is directly consumed on farm as green maize, with additional 25% retained by farmers for household consumption and other purposes (e.g., seed, gift and post-harvest losses). Non-human industrial use of maize is primarily driven by rising demand for poultry and aquaculture feed refueled by urbanization-induced expansion in demand for animal products. Maize makes up 60–65% of the ingredients in poultry feed and has thus been a major driver of its utilization in Nigeria (Dalberg, 2019). Current estimates suggest that maize utilization for animal feed accounts 30–40% of total national maize grain output (i.e., dry maize grain) (Dalberg, 2019). The poultry feed sector alone consumes up to 98% of all the animal feeds produced in the country, with just nine large poultry smallholder producers accounting for 70% of that consumption (CONTEXT, 2015).^7^

**Fig. 10:**
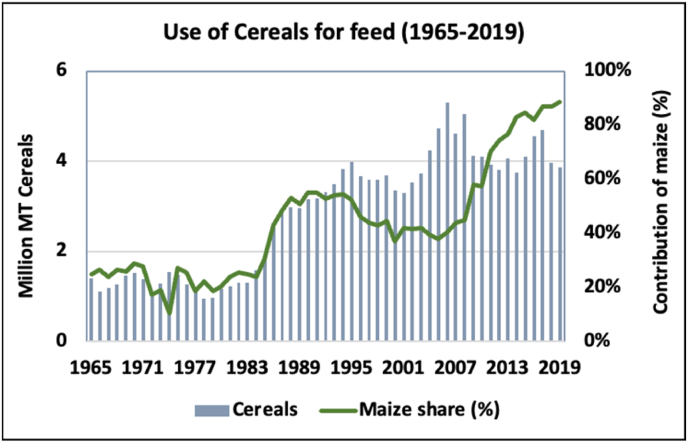
**Utilization of cereals and maize as feed**.

Source: Authors calculation based on FAOSTAT

Maize utilization trends for animal feed in Nigeria using the food balance sheets of the FAO (FAOSTAT, 2022) are presented in Fig. 10. The solid green line depicts the increasing rate in the utilization of maize as feed relative to other cereals. The contribution of maize to animal feed increased from about 20% in 1961 to 88% in 2020, with the growth rate accelerating in the last 20 years. The contribution of maize to cereal-based processed food products also increased from 20% to 26% during the same period. Available projections suggest that meat consumption in Nigeria is expected to double in the coming decades, which in turn is expected to further drive demand for maize as animal feed and for industrially processed maize-based food products (Dalberg, 2019; Erenstein et al., 2022).^8^

## 4 Conclusions and policy implications

In this paper, we documented the important changes that characterize Nigeria's maize production and area expansion over the last couple of decades. Using both secondary and primary data on maize production, productivity, and varietal adoption, we described the major drivers and mediating factors of the maize sector transformation in Nigeria. In this section, we provide additional insights with a focus on unexploited opportunities for further transformation of the maize sector of Nigeria along with implications for future research, policy, and public investment.

First, although impressive strides have been made in the development and promotion of improved maize varieties across diverse agro-ecological zones in Nigeria (Fajemisin, 2014; Badu-Apraku et al., 2018), there is still considerable potential for improving productivity with the increased use of modern inputs, including mechanization, irrigation, fertilizer, and improved seeds (Liverpool-Tasie et al., 2017; Abdoulaye et al., 2018). As less than 10% of the current maize production in Nigeria is irrigated and most maize farmers have limited access to site-specific and precision cropping system advisory services, the realization of genetic gains from the use of readily available improved maize varieties has been limited (Oyinbo et al., 2019, 2022). Therefore, well-targeted public investments, including digitally enabled advisory services for better and targeted extension advisory services can improve the re-organization of the maize value chain across the country, thereby improving the productivity, income, food, and nutritional security of maize farmers.

Second, despite substantial achievements in terms of increasing the production of certified maize seeds, accelerating varietal turnover, and reducing varietal age in farmers' fields, the potential demand for certified maize seed in Nigeria is still high and maize yields in the country remains at about 2 tons per ha and lags far below the yields achieved in other major maize-producing countries (FAOSTAT, 2022). The low productivity of maize in Nigeria is partly attributed to market imperfections that constrain farmers’ access to high-quality certified seeds of recently released improved maize varieties (Wossen et al., 2017a, Wossen et al., 2017b). Bold et al. (2017) reported that the productivity gain of replacing low-quality or adulterated hybrid maize seed with certified, first-generation (F1) hybrid seed in Uganda can reach 80%. Hence, one of the most viable pathways towards improving maize yields in the country is increasing the availability of certified seeds of improved maize varieties. As of 2021, the estimated maize area planted with certified improved maize seed was about 2.4 million hectares, accounting for 35% of the estimated maize area of 6.5 million ha in the country. This presents a 65% potential demand for certified maize seed in Nigeria. Nigeria could then become a major maize producer and exporter in the world through accelerated adoption of yield-enhancing and climate-resilient maize varieties released in recent years. To achieve this, policy interventions should be tailored to further scaling up of seeds of multiple stress tolerant maize varieties to build the resilience of smallholder farmers to climate shocks, increase maize productivity growth and improve the welfare of farming households.

A third opportunity is the development, dissemination, and adoption of hybrid maize seed. Both the certified seed production data and the DNA-fingerprinting based variety identification revealed that most of the improved maize varieties grown by farmers in Nigeria are OPVs. While conventional OPVs are generally regarded as less productive than hybrids, stress tolerant OPVs can withstand harsh environments, compared with local varieties, providing farmers with yield advantages (Abate et al., 2015, 2017). The high adoption rate of OPVs in the country has created awareness of the importance of good quality improved seeds among farmers that can trigger a shift from OPVs to hybrid seed. Although the seed-to-grain price ratio is about 8:1 for hybrids and 5:1 for OPVs, the greater yield advantage of hybrids over OPVs could incentivize farmers to replace OPVs with hybrids. Considerable efforts should thus be made to harness innovative tools, resources, and technologies for effective tackling of current and future challenges to accelerate the development, commercialization, and adoption of superior hybrids. This will also promote the establishment and consolidation of competitive private seed companies that market good quality seeds of stress resilient and productive hybrids on a sustainable basis.

Accelerating varietal turnover represents another potential intervention area for maximizing the impact of improved maize varieties in Nigeria. As breeding is largely an incremental process in which subsequent generations of maize varieties will be superior in grain yields, resistance to pests and disease, and nutritional quality (Abate et al., 2017; Ten Berge et al., 2019), farmers need to periodically replace old varieties with the new ones that deliver higher rates of genetic gains to their farms. Although Nigeria has made impressive progress in replacing old maize varieties with the new high-yielding and stress resilient maize varieties, further release, and commercialization particularly of hybrids with greater multiple stress tolerance and high yield potentials will ensure rapid productivity gains and production growth in the country. This requires strong support for introduction of innovative marketing strategies not only to get recently released varieties and new hybrids into the hands of smallholder farmers but also to accelerate varietal turnover, and ultimately increase the productivity and income of smallholder farmers. The emphasis on quality control and marketing of good quality certified seeds to farmers needs to be an integral part of the varietal replacement process. This is critical as the success of public investments in variety promotion depends not only on its ability to encourage a one-time switch from unimproved to improved varieties, but also on its ability to encourage a continuous or repeated process of varietal replacement by farmers. Along this line, understanding, and prioritizing farmers’ trait preferences would also be vital not only for proper targeting of the best suited maize varieties to farmers but also to help them derive the most benefit. As men and women farmers may differ in their valuation and preference of varietal attributes because of their different roles and responsibilities in seed choice, production, processing, consumption and marketing decisions, demand-driven breeding strategies that consider priority traits along gender, technical, market, social and environmental dimensions will thus be vital in packaging and delivering improved varieties that meet the diverse preferences of men and women farmers. A recent comparative analysis of seed demand among maize farmers in Nigeria (Bello et al., 2019) found a significantly higher willingness to pay for multiple stress-resilient maize varieties compared with varieties with tolerance to only drought or striga stress.

There is scope and opportunity for new research. First, research is needed to understand the varietal traits associated with greater adoption (e.g., tolerance to drought, tolerance to Striga, combined tolerance to drought and Striga, etc.) and their economic value (e.g., in terms of yield, variance and downside risk reduction). Second, rigorous research is needed to understand the commercial viability of various types of certified seed business and marketing strategies to better inform policymakers about effective and practical marketing strategies that could stimulate demand in an inclusive and cost-effective manner. Finally, it would be important to expand the evidence base on the sources of maize production and area growth in greater depth, especially with respect to quantifying and decomposing the contribution of technical change and demand-side drivers using detailed and dis-aggregated sub-national level data. Sub-national level analysis of land use changes across space and time would also be vital to better understand the sources of area expansion and the implications of this change for future land use.

## Declaration of competing interest

The authors declare that they have no known competing financial interests or personal relationships that could have appeared to influence the work reported in this paper.

## Figures and Tables

**Fig. A1:**
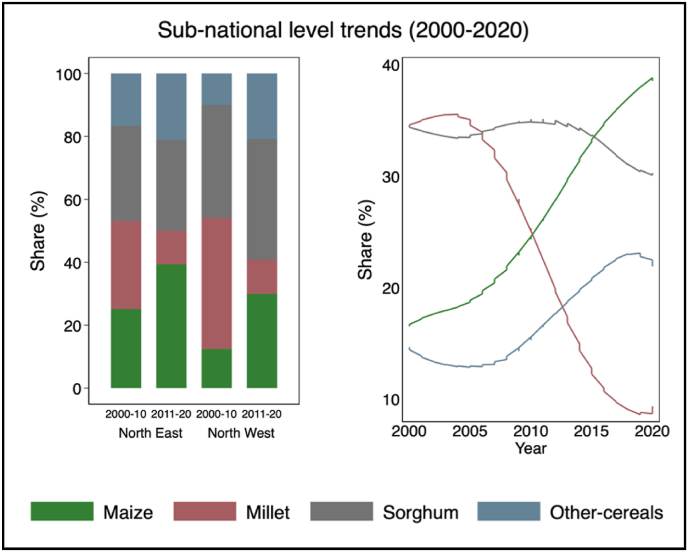
Additional sub-national level area replacement trends among competing cereal crops

**Fig. A2:**
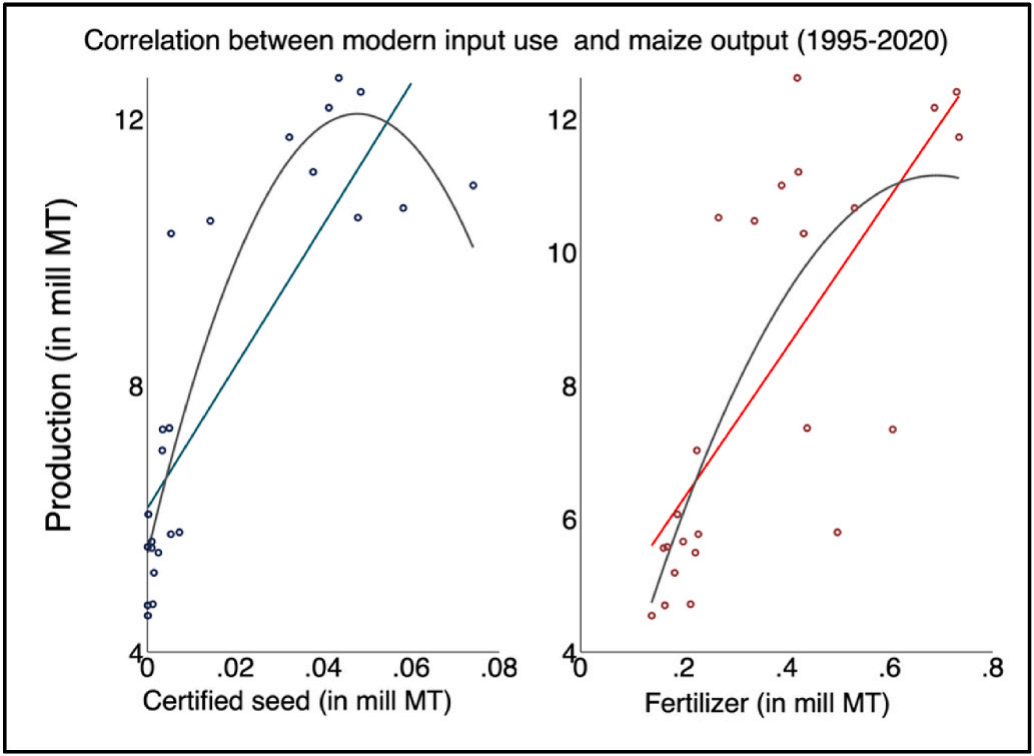
Country-level analysis: relationship between maize production and modern input use

**Fig. A3:**
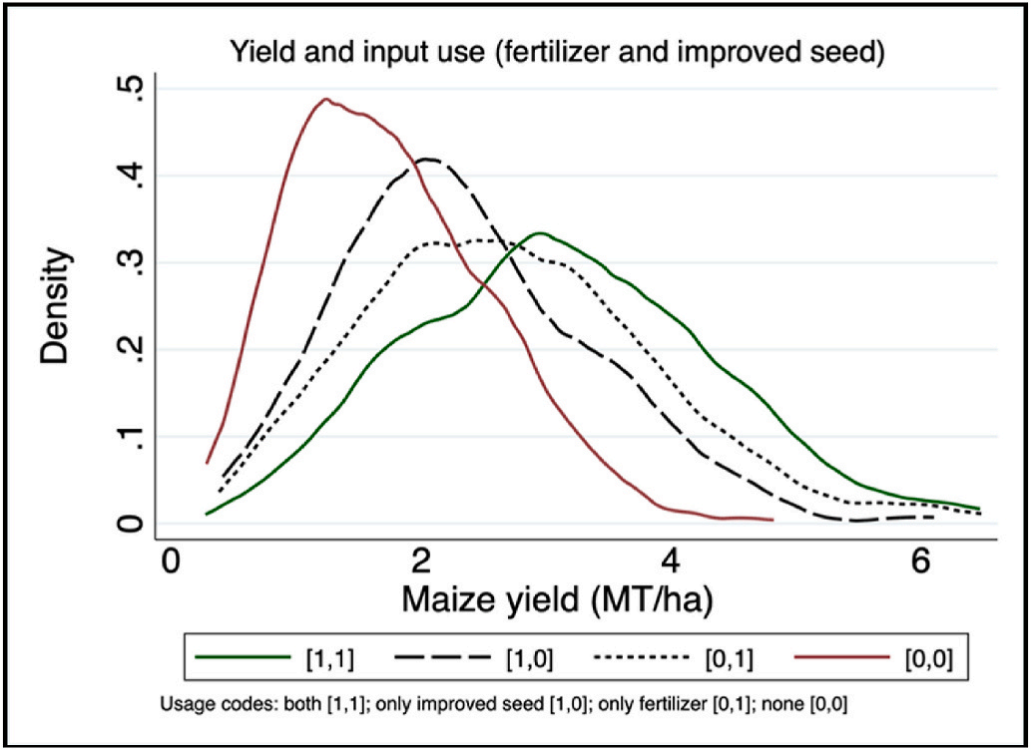
Micro-level analysis: non-parametric relationship between maize yield and modern input use

## Data Availability

Data will be made available on request.

## Footnotes

1 Additional sub-national and national level area and production changes over time are reported in Figure A1 in the appendix.

2 Herein, we focus on highlighting the key technical change and demand (market) side drivers of change simultaneously for which we had access to sufficient data. Identifying and decomposing the contributions of the specific mediating factors is beyond the scope of this paper.

3 As noted above, policy is not the only source/driver of maize production growth in the country as the maize sector transformation has been fostered by a combination of several factors, including input intensification, research efforts, extension, etc.

4 Formally registered in 2008, the Seed Entrepreneurs Association of Nigeria (SEEDAN) brings together seed companies and other key private sector players in the industry.

5 About 34% of the genotypes collected from the farmers' fields could not be matched to any of the samples in the reference library. Note that, some of the unidentified varieties could in fact be improved and hence we considered the 67% adoption rate based on the matched and identified varieties as a conservative lower bound estimate for Nigeria.

6 Additional correlation analysis between certified improved seed use and maize yields is reported in Fig A2 and A3 in the appendix.

7 The number of feed mills increased from 2 mills to over 1000 mills since 1957.

8 Note that, available evidence shows that the maize value chain currently creates close to 23 million jobs, mainly at the production level (i.e., for smallholder farmers who contributes more than 80% of total maize output of the country) and also among the growing number of micro/small-scale millers (Onumah et al., 2021).

## References

[bib1] Abate T., Shiferaw B., Menkir A., Wegary D., Kebede Y., Tesfaye K., Kassie M., Bogale G., Tadesse B., Keno T. (2015). Factors that transformed maize productivity in Ethiopia. Food Sec.

[bib2] Abate T., Fisher M., Abdoulaye T., Kassie G.T., Lunduka R., Marenya P., Asnake W. (2017). Characteristics of maize cultivars in Africa: how modern are they and how many do smallholder farmers grow?. Agric. Food Secur..

[bib3] Abdoulaye T., Wossen T., Awotide B. (2018). Impacts of improved maize varieties in Nigeria: ex-post assessment of productivity and welfare outcomes. Food Secur..

[bib4] Aggarwal S., Giera B., Jeong D., Robinson J., Spearot A. (2018).

[bib5] Alene A.D., Menkir A., Ajala S.O., Badu-Apraku B., Olanrewaju A.S., Manyong V.M., Ndiaye A. (2009). The economic and poverty impacts of maize research in West and Central Africa. Agric. Econ..

[bib6] Badu‐Apraku B., Fakorede M.A.B., Oyekunle M., Yallou G.C., Obeng‐Antwi K., Haruna A., Akinwale R.O. (2015). Gains in grain yield of early maize cultivars developed during three breeding eras under multiple environments. Crop Sci..

[bib7] Badu-Apraku B., Talabi A.O., Ifie B.E., Chabi Y., Obeng-Antwi K., Haruna A., Asiedu R. (2018). Gains in grain yield of extra-early maize during three breeding periods under drought and rain-fed conditions. Crop Sci..

[bib8] Bello M., Abdoulaye T., Abdulai A., Wossen T., Menkir A. (2019).

[bib52] Byerlee D. (2020). The globalization of hybrid maize, 1921–70. J. Glob. History.

[bib9] Byerlee D., Jewell D., Byerlee D., Eicher C.K. (1997). Africa's Emerging Maize Revolution.

[bib10] Byerlee D., Stevenson J., Villoria N. (2014). Does intensification slow crop land expansion or encourage deforestation?. Global Food Secur..

[bib11] Bold T., Kaizzi K.C., Svensson J., Yanagizawa-Drott D. (2017). Lemon technologies and adoption: measurement, theory and evidence from agricultural markets in Uganda. Q. J. Econ..

[bib12] Chivasa W., Worku M., Teklewold A., Setimela P., Gethi J., Magorokosho C., Prasanna B.M. (2022). Maize varietal replacement in Eastern and Southern Africa: bottlenecks, drivers and strategies for improvement. Global Food Secur..

[bib13] CONTEXT (2015).

[bib14] Dalberg (2019).

[bib15] Erenstein O., Jaleta M., Sonder K., Mottaleb K., Prasanna B.M. (2022). Global maize production, consumption and trade: trends and R&D implications. Food Secur..

[bib16] Evenson R.E., Gollin D. (2003). Assessing the impact of the green revolution, 1960 to 2000. Science.

[bib17] Fajemisin J.M. (2014).

[bib18] Fakorede M.A.B., Ajala S.O., Fajemisin J.M. (2022).

[bib19] Fisher M., Abate T., Lunduka R.W., Asnake W., Alemayehu Y., Madulu R.B. (2015). Drought tolerant maize for farmer adaptation to drought in sub-Saharan Africa: determinants of adoption in eastern and southern Africa. Climatic Change.

[bib20] Foltz J.D., Aldana U.T., Laris P. (2012).

[bib21] Gollin D., Hansen C.W., Wingender A.M. (2021). Two blades of grass: the impact of the green revolution. J. Polit. Econ..

[bib22] Kassam A.H., Kowal J., Dagg M., Harrison M.N. (1975). Maize in West Africa: and its potential in savanna areas. World Crop..

[bib23] Liverpool-Tasie L.S.O., Omonona B.T., Sanou A., Ogunleye W.O. (2017). Is increasing inorganic fertilizer use for maize production in SSA a profitable proposition? Evidence from Nigeria. Food Pol..

[bib24] Mabaya E., Ajayi S.A., Waithaka M., Tihanyi K., Mugoya M., Kanyenji G. (2021).

[bib25] McCann J.C. (2005).

[bib26] Miracle M.P. (1965). The introduction and spread of maize in Africa. J. Afr. Hist..

[bib27] NACGRAB (2014).

[bib32] NBS (2007).

[bib33] NBS (2011).

[bib34] Olomola A.S. (2015).

[bib35] Onumah G., Dhamankar M., Ponsioen T., Bello M. (2021).

[bib36] Oyinbo O., Mbavai J.J., Shitu M.B., Abdoulaye T., Ugbabe O.O. (2019). Sustaining the beneficial effects of maize production in Nigeria: does adoption of short season maize varieties matter?. Exp. Agric..

[bib37] Oyinbo O., Chamberlin J., Abdoulaye T., Maertens M. (2022). Digital extension, price risk and farm performance: experimental evidence from Nigeria. Am. J. Agric. Econ..

[bib38] Pwc (2021). https://www.pwc.com/ng/en/assets/pdf/positioning-nigeria-as-africa-leader-in-maize-production-for-afcfta.pdf.

[bib39] Rutsaert P., Chamberlin J., Oluoch K.O.A., Kitoto V.O., Donovan J. (2021). The geography of agricultural input markets in rural Tanzania. Food Secur..

[bib40] Sheahan M., Barrett C.B., Sheahan M.B. (2014).

[bib41] Simtowe F., Amondo E., Marenya P., Rahut D., Sonder K., Erenstein O. (2019). Impacts of drought-tolerant maize varieties on productivity, risk, and resource use: evidence from Uganda. Land Use Pol..

[bib42] Smith J., Barau A.D., Goldman A., Mareck J.H. (1994). The role of technology in agricultural intensification: the evolution of maize production in the Northern Guinea Savanna of Nigeria. Econ. Dev. Cult. Change.

[bib43] Smith J., Weber G., Manyong M.V., Fakorede M.A.B. (1997).

[bib44] Ten Berge H.F., Hijbeek R., Van Loon M.P., Rurinda J., Tesfaye K., Zingore S., van Ittersum M.K. (2019). Maize crop nutrient input requirements for food security in sub-Saharan Africa. Global Food Secur..

[bib45] van Ittersum M.K., van Bussel L.G.J., Wolfa J., Grassini P., van Wart J., Guilpart N., Claessens L., de Groot H., Wieb K., Mason-D’Croz D., Yang H., Boogaard H., van Oort P.J.A., van Loon M.P., Saito K., Adimoh O., Adjei-Nsiah S., Agali A., Bala A., Chikowo R., Kaizzi K., Kouressy M., Makoi J.H.J.R., Ouattara K., Tesfaye K., Cassman K.G. (2016). Can sub-Saharan Africa feed itself?. Proc. Natl. Acad. Sci. USA.

[bib46] Waithaka M., Mugoya M., Ajayi A., Okelola F., Tihanyi K. (2019).

[bib47] Wineman A., Njagi T., Anderson C.L., Reynolds T.W., Alia D.Y., Wainaina P., Njue E., Biscaye P., Ayieko M.W. (2020). A case of mistaken identity? Measuring rates of improved seed adoption in Tanzania using DNA fingerprinting. J. Agric. Econ..

[bib48] Wossen T., Abdoulaye T., Alene A., Feleke S., Ricker-Gilbert J., Manyong V., Awotide B.A. (2017). Productivity and welfare effects of Nigeria's e-voucher-based input subsidy program. World Dev..

[bib49] Wossen T., Abdoulaye T., Alene A., Feleke S., Menkir A., Manyong V. (2017). Measuring the impacts of adaptation strategies to drought stress: the case of drought tolerant maize varieties. J. Environ. Manag..

[bib50] Wossen T., Abdoulaye T., Alene A., Nguimkeu P., Feleke S., Rabbi I.Y., Haile M.G., Manyong V. (2019). Estimating the productivity impacts of technology adoption in the presence of misclassification. Am. J. Agric. Econ..

[bib51] Wossen T., Abay K.A., Abdoulaye T. (2022). Misperceiving and misreporting input quality: implications for input use and productivity. J. Dev. Econ..

[bib55] FAOSTAT. (2022). Statistical databases and data-sets of the Food and Agriculture Organization of the United Nations. http://faostat.fao.org/default.aspx. Accessed August 2023.

